# The choice of cryopreservation method affects immune compatibility of human cardiovascular matrices

**DOI:** 10.1038/s41598-017-17288-z

**Published:** 2017-12-05

**Authors:** Maria Schneider, Christof Stamm, Kelvin G. M. Brockbank, Ulrich A. Stock, Martina Seifert

**Affiliations:** 1 Institute of Medical Immunology, Charité - Universitätsmedizin Berlin, corporate member of Freie Universität Berlin, Humboldt-Universität zu Berlin, and Berlin Institute of Health, Berlin, Germany; 2 Berlin-Brandenburg Center for Regenerative Therapies (BCRT), Charité - Universitätsmedizin Berlin, corporate member of Freie Universität Berlin, Humboldt Universität zu Berlin, and Berlin Institute of Health, Berlin, Germany; 3 German Heart Center (DHZB), Charité - Universitätsmedizin Berlin, Berlin, Germany; 4 Tissue Testing Technologies LLC, North Charleston, SC USA; 5 Department of Regenerative Medicine and Cell Biology, Medical University of South Carolina, Charleston, SC USA; 6 Royal Brompton and Harefield NHS Trust Imperial College London, London, UK

## Abstract

Conventional frozen cryopreservation (CFC) is currently the gold standard for cardiovascular allograft preservation. However, inflammation and structural deterioration limit transplant durability. Ice-free cryopreservation (IFC) already demonstrated matrix structure preservation combined with attenuated immune responses. In this study, we aim to explore the mechanisms of this diminished immunogenicity *in vitro*. First, we characterized factors released by human aortic tissue after CFC and IFC. Secondly, we analyzed co-cultures with human peripheral blood mononuclear cells, purified monocytes, T cells and monocyte-derived macrophages to examine functional immune effects triggered by the tissue or released cues. IFC tissue exhibited significantly lower metabolic activity and release of pro-inflammatory cytokines than CFC tissue, but surprisingly, more active transforming growth factor β. Due to reduced cytokine release by IFC tissue, less monocyte and T cell migration was detected in a chemotaxis system. Moreover, only cues from CFC tissue but not from IFC tissue amplified αCD3 triggered T cell proliferation. In a specifically designed macrophage-tissue assay, we could show that macrophages did not upregulate M1 polarization markers (CD80 or HLA-DR) on either tissue type. In conclusion, IFC selectively modulates tissue characteristics and thereby attenuates immune cell attraction and activation. Therefore, IFC treatment creates improved opportunities for cardiovascular graft preservation.

## Introduction

Providing matrices for tissue replacements that fulfill all requirements for successful and appropriate treatment of cardiovascular diseases is still an important issue. The ability to replace tissues on demand would help to decrease the mortality rate in many clinical scenarios. Off-the-shelf available vascular grafts and heart valves for example would be needed for bypass surgery^1,2^, cardiac valvular pathologies including rheumatic fever, atherosclerosis in elderly patients or congenital malformation in children^3–6^, shunts for dialysis patients^7,8^ and vessel reconstruction after organ transplantation^9,10^. Therefore, improved strategies and techniques for tissue preservation have to be developed to tackle this urgent and so far unmet medical need^11^.

Biological vascular and heart valve matrices appear to be beneficial compared to synthetic or mechanical substitutes with respect to hemodynamic features, biocompatibility, anti-coagulation treatment, and their potential to grow and remodel especially in young patients^4,6,12^. However, the availability of human biological matrices for an off-the-shelf allogeneic application is limited. Therefore, xenogeneic (porcine and bovine) tissue is often used for heart valve replacement. To avoid unwanted xeno-immune responses, the tissue is fixed with glutaraldehyde (GA). However, after fixing, the tissue is not viable and lacks remodeling capacity^13,14^. Another treatment option to reduce immune responses to xenogeneic tissue following implantation is the introduction of a decellularization step that lowers or even eliminates potential immune response triggers, e.g. foreign cells or debris^15–20^. However, reduction of immunogenicity by implementing decellularization is achieved to date at the risk of altering the structure of the extracellular matrix (ECM) and represents a cost and time intensive method^21,22^.

In terms of immunogenicity, allogeneic tissue has some advantages over xenogeneic tissue. Allografts, also known as homografts, can be preserved and stored without the need for complex and long lasting pre-treatment procedures by conventional frozen cryopreservation (CFC). Allografts for heart valve replacement showed good long-term performance and higher durability^23,24^. The CFC method is the most commonly used method of human heart valve cryopreservation in the clinic. Allografts are frozen in cell culture medium supplemented with serum and dimethyl sulfoxide (DMSO) at a controlled rate of −1 °C and stored in the vapor phase above liquid nitrogen. However, detectable ice-crystal formation in those tissues might enhance the release of soluble factors and cellular components that are able to trigger calcification processes *in vivo* and lead to deterioration of the tissue^25,26^.

To prevent ice-crystal formation in cardiovascular tissues and to reduce the associated higher risk for calcification, an alternative ice-free cryopreservation (IFC) method was developed, which promotes ECM retention and loss of cell viability^27^. The IFC method was developed from heart valve vitrification studies. In the process of up-scaling to full-sized heart valve allografts the initial vitrification solution (VS) was modified, by increasing the concentration of the three cryoprotectants 1,2-propanediol, formamide, and DMSO to 83%. The new formulation, which was designated “VS83”, was potentially stable above its glass transition temperature at −80 °C. Therefore, storage at −80 °C was subsequently incorporated into the IFC method, which would make it easier and cheaper to store and ship the tissue samples^27^. Also, the single step cryoprotectant loading at room temperature and the thawing and washout protocol differs from typical vitrification protocols. The evolution of the IFC process employed here has been reviewed in depth^28^. The improved protocol with VS83 was already successfully applied to cardiovascular material and demonstrated better preservation of the ECM structure^29,30^. Accordingly, in an allogeneic sheep model it could be shown that this preservation method resulted in better performance, with less thickening of heart valve tissue and reduced immune cell infiltration after *in vivo*-implantation compared to conventional frozen grafts^31,32^.

Recently published data from our group demonstrated reduced immune cell responses including T cell proliferation and cytokine release in co-cultures with ice-free cryopreserved xenogeneic heart valve matrices *in vitro* ^33^. A post-thaw-treatment of human heart valves only with the VS83 solution demonstrated an improved immune compatibility^34^, but the effect of IFC on the immunogenicity of human cardiovascular tissue has not been analyzed so far.

It is currently unknown why the IFC method is able to reduce the immune responses against cardiovascular tissue *in vitro* as well *in vivo*. Moreover, it would be of importance to know whether the specifically ice-free cryopreserved tissue exhibits an immune modulatory capacity. This is essential because immune responses after tissue implantation pass through different phases, starting with protein adsorption and leading to infiltration of innate immune cells^35^. Infiltration of monocytes into the tissue is one of the first steps of inflammation but is also known to be necessary for a subsequently beneficial remodeling process. Depending on the tissue milieu or environmental cues, monocyte-derived macrophages with different activation and polarization features develop and represent distinct functional phenotypes^36–38^. Predominantly inflammatory signals within the ECM would favor the development of classical M1-type macrophages, whereas signals favoring tissue repair would promote the generation of alternative M2-type macrophages^39–41^. However, it is also possible that macrophages display various polarization stages and mixed macrophage phenotypes or even a reversion of the polarization are frequently observed^38,42^.

In the present study, we use human aortic tissue as a “proof of concept” material for heart valve allografts and vascular grafts. The CFC method preserves cell viability at the expense of the ECM, while the IFC method preserves ECM at the expense of cell viability^43^. We aimed to characterize the tissue properties of IFC tissue in comparison to the CFC matrices in detail, thereby analyzing features that impact immunological compatibility like viability, metabolic activity and release of cytokines and chemokines. Furthermore, early and late immune response events were analyzed by determining the monocyte migration, the adherence, activation and polarization behavior of human monocyte-derived macrophages as well as the impact on T cell attraction and proliferation.

In this study, we provide conclusive results to understand the mechanisms responsible for a diminished initiation of immune responses by IFC tissue in an allogeneic setting as a precondition for potential clinical application.

## Results

### CFC and IFC aortic tissue show similar histological but altered metabolic features

**Figure 1:**
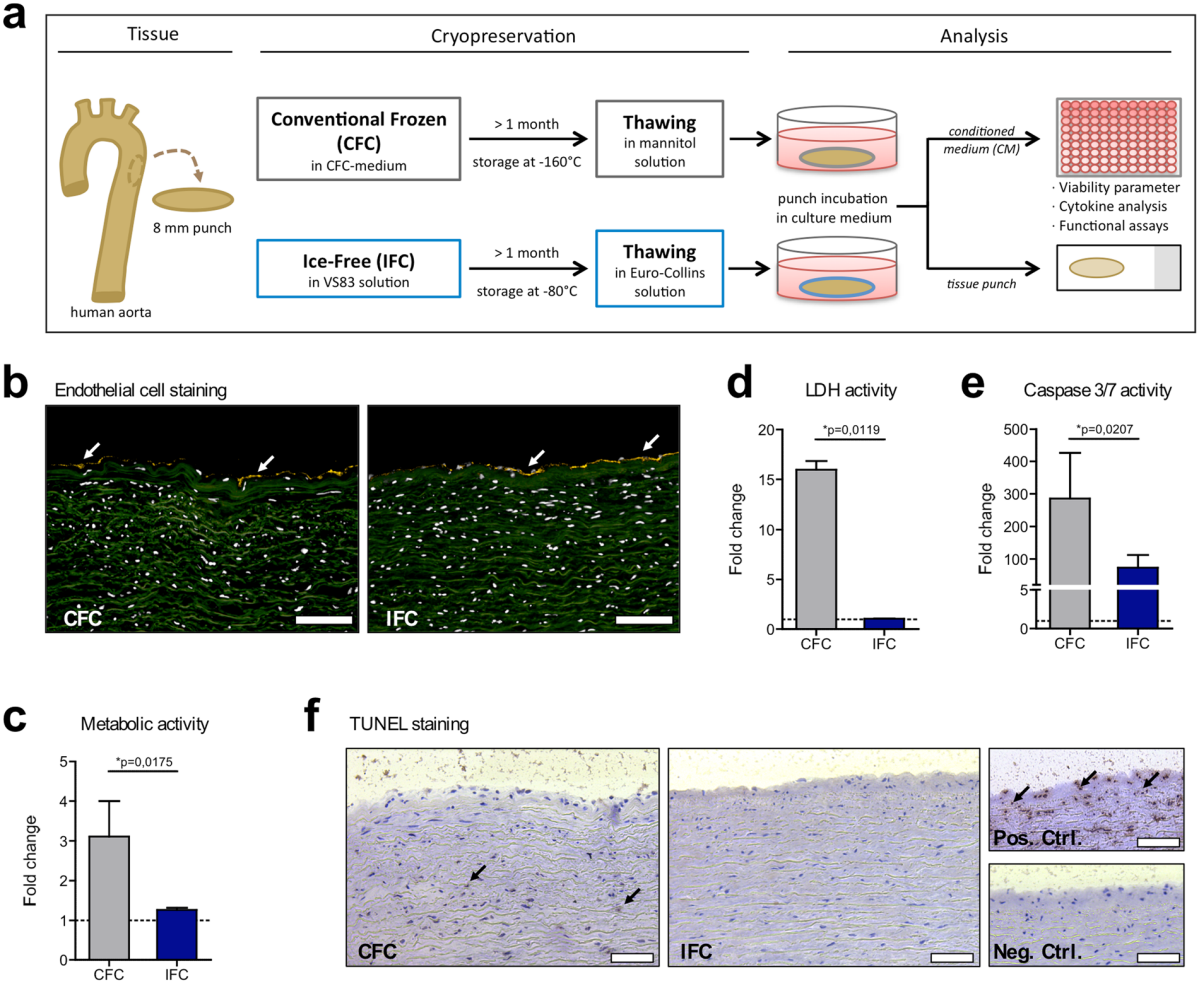
Histological and metabolic characteristics of human aortic tissue after Conventional Frozen Cryopreservation (CFC) or Ice-Free Cryopreservation (IFC). (**a)** The tissue preparation and analysis is shown schematically. Punches of fresh aortic tissue were made and frozen according to either the CFC or IFC protocols (see methods). Tissue punches were stored at least 1 month in the vapor phase above liquid nitrogen (about −160 °C) or at −80 °C before use, respectively. Tissue samples were thawed and washed according to the CFC or IFC protocols for further analysis. Punches were incubated in cell culture medium and tissue viability (metabolism, apoptosis, and necrosis) and cytokine release were analyzed, and tissue specimens were histologically examined. (**b)** Human CD31+ staining (yellow) indicates the endothelial cell layer (white arrows) on the intimal side of both CFC and IFC aortic tissue. Nuclei were stained with DAPI (pseudo-colored white); while the extracellular matrix shows green autofluorescence. Scale bars represent 100 µm. (**c)** Metabolic activity of the tissue was assessed with an MTS assay. (**d)** Necrosis level was determined by the measurement of lactate dehydrogenase (LDH) activity in conditioned medium (CM). (**e)** Apoptosis of tissue cells was analyzed via measurement of caspase-3 and -7 activities in the CM. All measurements are normalized to the medium control (dotted line). Data are shown as the mean + SEM (n = 5–7) and analyzed with Mann Whitney test *p < 0.05. (**f)** TUNEL staining revealed some apoptotic cells (brown nuclei) highlighted by black arrows in the CFC tissue. The DNAse-treated positive control (Pos. Ctrl.), and a negative control (Neg. Ctrl.) are shown for comparison. Scale bars represent 75 µm.

In the present study, the influence of the cryopreservation technique on the immunogenicity of cardiovascular tissue was evaluated. We compared the conventional frozen cryopreservation (CFC), which is a commonly used protocol in the clinic, with the ice-free cryopreservation (IFC), following a newly developed vitrification protocol (see more details to the underlying preservation concept in Supplementary information). We used human aortic tissue as a “proof of concept” material for heart valve allografts and vascular grafts. Biopsy punches of aortic tissue were obtained and frozen according to the CFC or IFC protocols (Fig. 1a). After storage for at least 1 month, histological characteristics of the thawed tissues were assessed. Moreover, metabolic features of the tissues were analyzed, including released factors (conditioned medium) and their influence on immune cell activation.

To examine the structure of CFC and IFC aortic tissues, cryosections were stained for the endothelial cell marker CD31. The endothelial layer with distinct CD31+ cells on the intimal side of the aortic tissue was still present after using either cryopreservation method (Fig. 1b). Nuclear counterstaining with DAPI showed that no decellularization occurred after either treatment. Metabolic activity after freezing and thawing was measured using the MTS assay. CFC tissue revealed a high metabolic activity, whereas IFC tissue showed minimal metabolism (Fig. 1c). Assuming that cell death occurred during freezing, necrotic and apoptotic events were analyzed. To first investigate the number of necrotic cells with damaged cell membranes, the lactate dehydrogenase (LDH) released from the tissue was measured. Surprisingly, high LDH activity was detected in CFC tissue, but not in IFC tissue (Fig. 1d). Apoptosis was then assessed via caspase measurement of the supernatant and histologically by TUNEL (TdT-mediated dUTP-biotin nick end labeling) staining. Also, significantly higher levels of Caspase 3/7 activity, representing early apoptotic events, were measured in CFC tissue compared to IFC tissue (Fig. 1e). TUNEL staining was also positive for CFC tissue, confirming that some cells had DNA fragmentation indicating apoptosis (Fig. 1f).

### CFC and IFC aortic tissue display different cytokine and chemokine profiles

**Figure 2:**
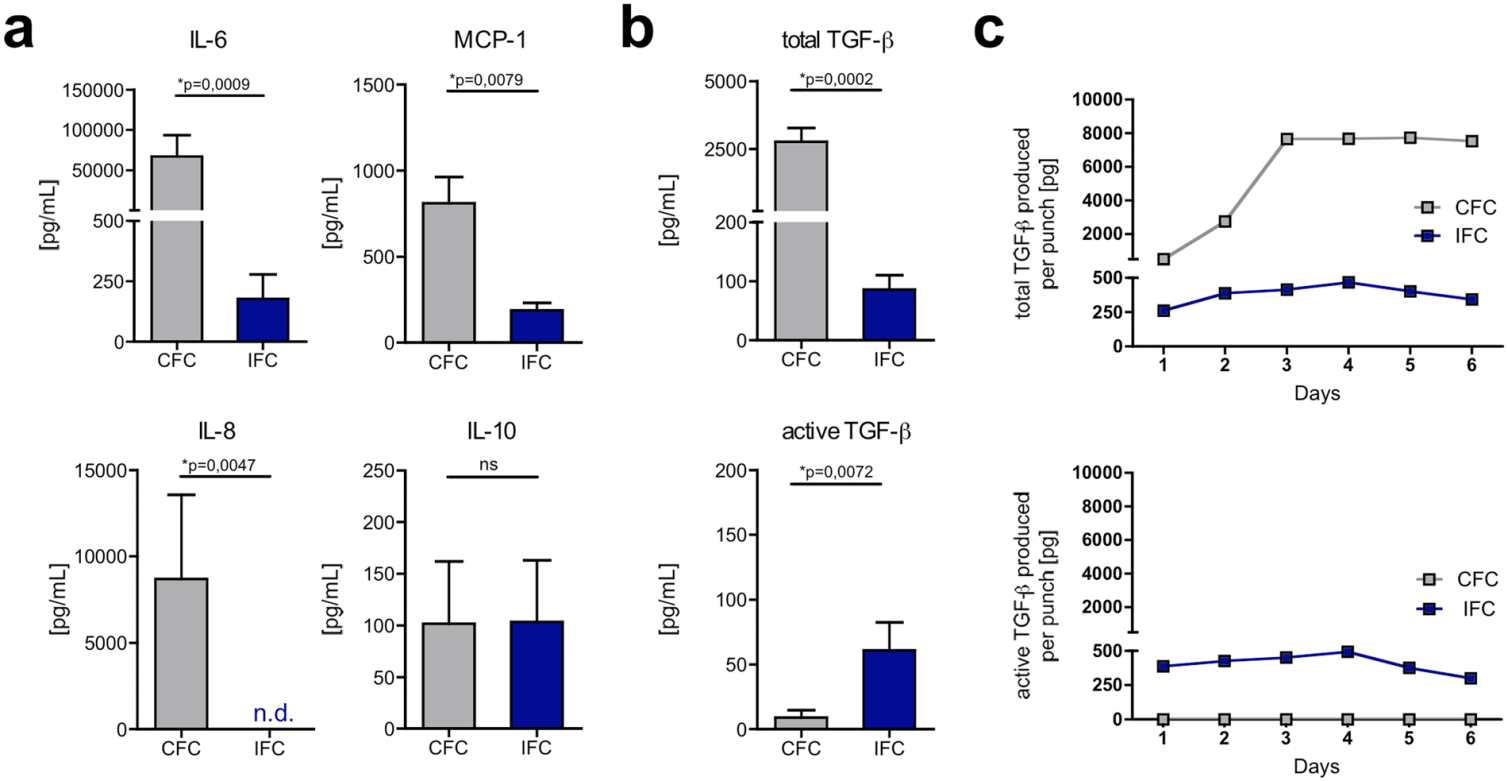
IFC tissue releases lower cytokine and chemokine levels than CFC tissue, however more biologically active TGF-β is present. CFC and IFC aortic tissue punches were incubated in DMEM culture medium for 2 days. The cytokines and chemokines IL-6, MCP-1 IL-8, IL-10 **(a)** total TGF-β (active and latent form) and active TGF-β **(b)** were analyzed by ELISA or multiplex bead assay. Data are shown as the mean + SEM (n = 4–8) and analyzed with Mann Whitney test **p < 0.01, ***p < 0.01; n.s.: not significant; n.d.: not detectable. (**c**) Over a culture period of 6 days, total TGF-β (upper graph) and active TGF-β (lower graph) was measured by ELISA. The absolute amount of TGF-β in pg produced per tissue punch was calculated and a representative kinetic graph is shown.

To assess the immunomodulatory potential of CFC and IFC tissues, cytokines and chemokines released from the tissues were analyzed by ELISA and multiplex bead assay (Fig. 2). After 2 days of tissue culture, significantly more IL-6, MCP-1 and IL-8 was released by CFC compared to IFC tissue. IL-10 was released in equal amounts from both tissues (Fig. 2a). It should be noted that levels of TNF-α, IFN-γ, IL-1β and IL-12p70 were also measured, but were below 5 pg/mL or not detectable (data not shown). Moreover, TGF-β was determined as both the latent (inactive) and the biologically active form. The amounts of total TGF-β (latent and active form) and exclusively of active TGF-β are shown in Fig. 2b. Similarly, to most of the other cytokines measured, significantly more total TGF-β was detected in conditioned medium from CFC tissue compared to IFC tissue medium. However, when only looking at the proportion of active TGF-β related to total TGF-β, it was higher for IFC tissue compared to CFC. Considering the actual amounts, it can be concluded that most of the total TGF-β released from IFC tissue was in its biologically active form, whereas CFC tissue released predominantly the latent form. To discriminate between active and total TGF-β, an acidification step was necessary in the TGF-β ELISA to activate the bound inactive form of TGF-β in the sample. Instead of performing this acidification step, we treated the CFC conditioned medium with the components of the CFC freezing medium and the VS83 (IFC) solution. With 10% DMSO, as is contained in CFC freezing medium, 1% of the latent TGF-β could be activated. However, with either the 36% DMSO or 21% formamide (components of the VS83 solution), 16% and 8%, respectively, were activated (Supplementary Fig. S1). Next, we analyzed the total amount of TGF-β accumulated in the tissue culture over 6 days (Fig. 2c). The absolute amount of total TGF-β produced per tissue punch in the CFC tissue culture increased in the first 3 days, and then remained at that level. The amount of total TGF-β was generally lower in IFC tissue cultures than in CFC samples, and did not dramatically increase over time. The same characteristics were seen for active TGF-β in IFC tissue culture, while active TGF-β levels were minimal for CFC tissue cultures (Fig. 2c). The accumulation of IL-6 and MCP-1 over the 6 days was also measured, and it was found that CFC tissue released a constant amount of these cytokines, while IFC tissue levels were low (see Supplementary Fig. S2).

### Immune cell migration is not induced by IFC aortic tissue

**Figure 3:**
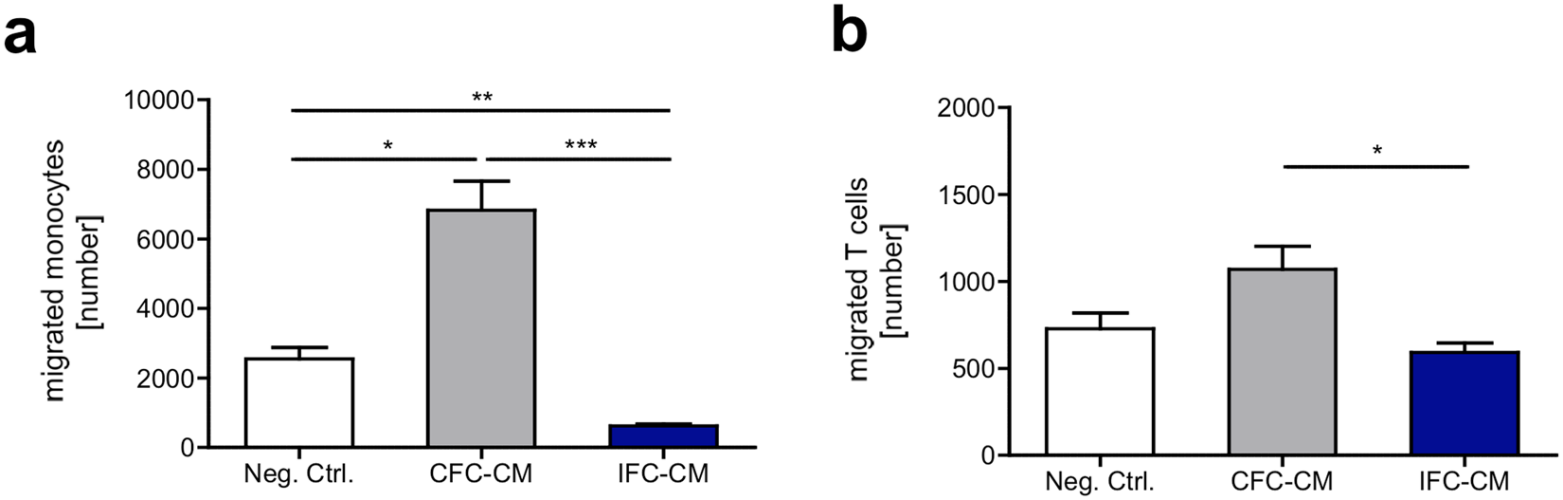
Immune cell migration is triggered by soluble factors delivered by CFC tissue, but not IFC tissue. Monocytes (CD14+) and T cells (CD3+) were separated from human PBMC. Thirty thousand cells in diet-medium were seeded on the porous membrane of a chemotaxis system. CFC or IFC tissue conditioned diet-medium (CM) was placed in the lower well. After 3 h, the number of migrated monocytes **(a)** and T cells **(b)** was analyzed. Migrated cells were defined as cells in the lower well plus cells attached to the bottom side of the membrane. Pure diet-medium served as negative control (Neg. Ctrl.), to define the random cell migration without a chemotactic gradient. Data are shown as the mean + SEM (n = 5, 3 replicates each) and analyzed with one-way ANOVA (Kruskal-Wallis test) *p < 0.05, **p < 0.01, ***p < 0.001.

Subsequently we determined whether the differential release of soluble factors by CFC and IFC tissue influenced the migratory behavior of immune cells (Fig. 3). Purified human monocytes and T cells were seeded in diet-medium onto the porous membrane of a chemotaxis system. CFC or IFC tissue conditioned medium (CM) that was previously generated from 6-day cultures served as attractants on the other side of the membrane. After 3 hours, the cells that migrated into the lower well or which were attached to the bottom side of the membrane were counted. In general, monocytes showed a higher migratory activity than T cells. CFC-CM attracted significantly more monocytes (Fig. 3a) and T cells (Fig. 3b) than IFC-CM. The number of migrated monocytes and T cells towards the IFC-CM was equal to or even less than the negative control. Migrated cells in the negative control indicate the level of non-directed, random cell migration without any chemotactic gradient. In the control settings, significantly more monocytes migrated towards the supernatant from an αCD3/CD28 stimulated PBMC culture (positive control) and medium containing IL-6 than to the negative control (Supplementary Fig. S3a). The controls for T cells also showed a trend of higher migration towards the positive control, but differences were not significant (Supplementary Fig. S3b).

### T cell proliferation is enhanced by CFC aortic tissue

**Figure 4:**
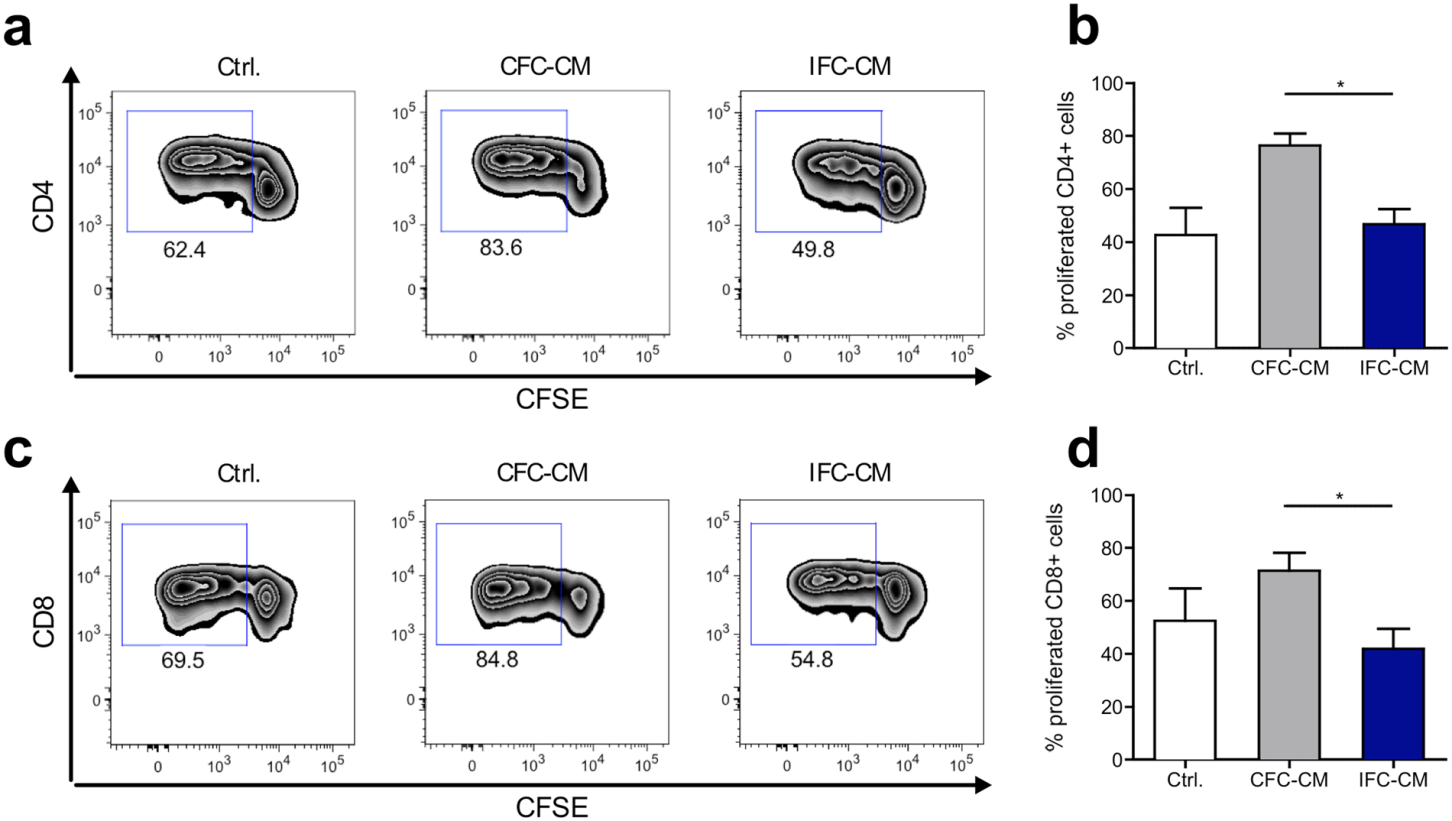
Soluble factors from CFC tissue but not from IFC tissue amplify αCD3 triggered T cell proliferation. Human CFSE-labeled PBMC were stimulated with a low dose of αCD3 antibody and subjected to CFC or IFC tissue conditioned medium (CM). After 4 days, PBMC were harvested, stained with fluorochrome-labeled human specific antibodies for T cell subset markers and analyzed by flow cytometry. Representative flow cytometry plots show the induction of CD4+ T cell **(a)** and CD8+ T cell **(c)** proliferation in PBMC cultures stimulated with either αCD3 alone (Ctrl.; left), or combined with CFC tissue CM (CFC-CM; middle) or IFC tissue CM (IFC-CM; right). Bar graphs show the levels of proliferated CD4+ T cells **(b)** and CD8+ T cells **(d)** in summary. Data are shown as the mean + SEM (n = 5–8) and analyzed with one-way ANOVA (Kruskal-Wallis test) *p < 0.05.

To predict adaptive response mechanisms, we evaluated whether just the soluble factors released by CFC and IFC aortic tissue could induce or modulate T cell responses. Immune cell cultures (PBMC) were treated with CFC tissue or IFC tissue CM. Without any additional triggers, T cells in the PBMC culture remained inactivated and did not proliferate (Supplementary Fig. S4a). Since T cell activation requires two signals, we then provided the first signal by low dose stimulation with αCD3, and analyzed whether the CM was able to deliver a second co-stimulatory signal that would enhance the immune cell response. After 4 days of stimulation, PBMC were harvested and analyzed by flow cytometry. After applying a gating strategy to define viable T cells (Supplementary Fig. S4b), T cell proliferation was determined based on the dilution of the CFSE fluorescence signal. CM from CFC tissue induced significantly more CD4+ (Fig. 4a,b) and CD8+ (Fig. 4c,d) T cell proliferation than CM from IFC tissue. IFC tissue CM did not alter either the CD4+ or CD8+ T cell responses compared to the control.

### Macrophage response to cryopreserved human aortic tissue

**Figure 5:**
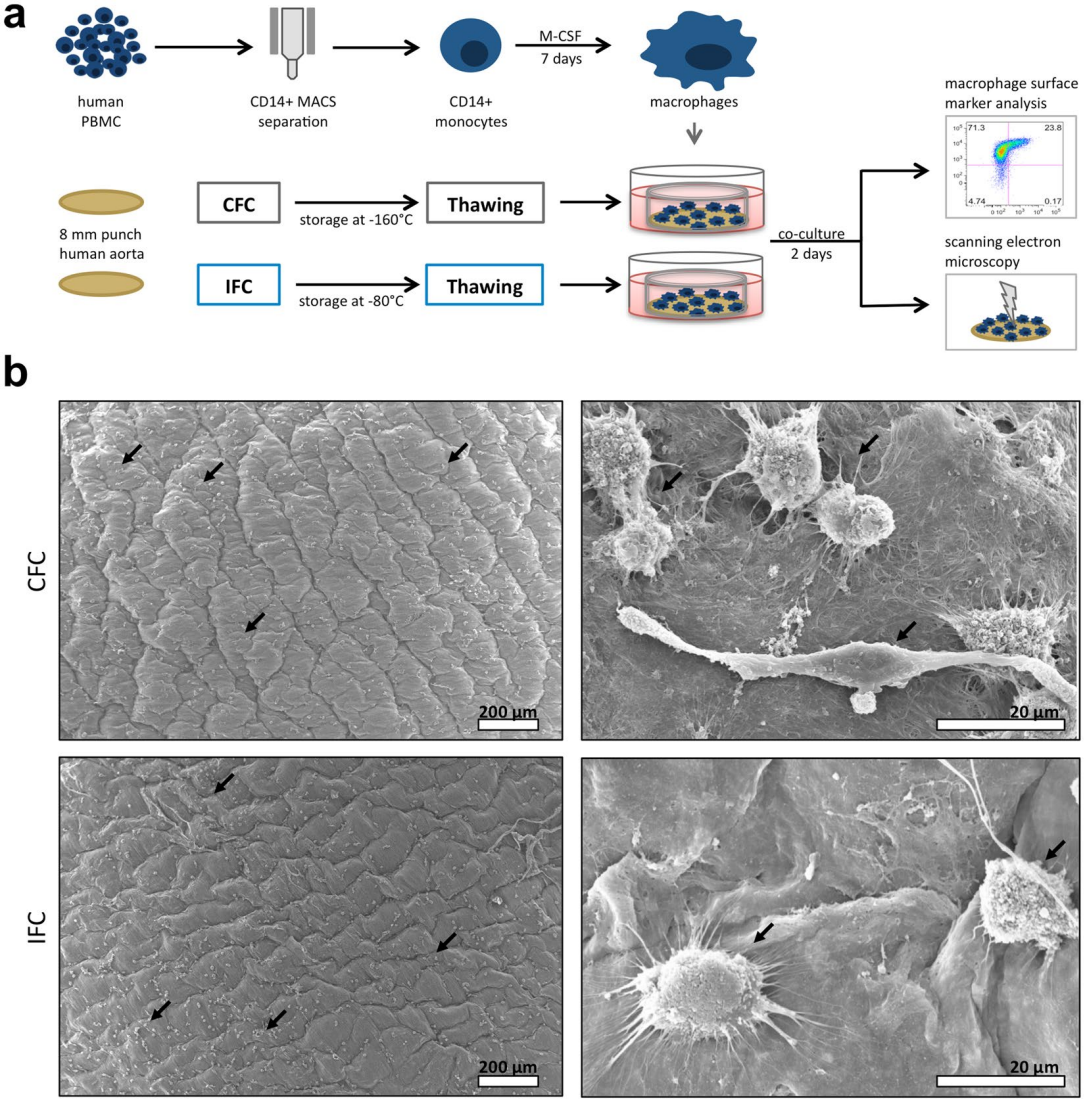
Macrophages cultured on CFC or IFC tissue show comparable adherence and appearance. (**a)** In a newly developed macrophage-tissue assay, macrophages were cultured directly on the aortic tissue surface. Monocytes were separated from human PBMC with MACS CD14 MicroBeads. Monocytes were differentiated to macrophages *in vitro* for 7 days with M-CSF and seeded directly on the surface (intimal side) of the human CFC or IFC treated aorta. A silicone ring held the tissue punch on the bottom of the culture well to ensure direct contact of macrophages and tissue. After a 2-day co-culture, the tissue punch was either analyzed with scanning electron microscopy (SEM), or the macrophages were harvested and their surface marker expression pattern was analyzed by flow cytometry. (**b)** Representative SEM pictures of the macrophage-tissue co-culture are depicted. The intima surface of CFC (upper row) and IFC (lower row) aortic tissue are shown with macrophages adhered to the tissue surface (black arrows). In higher magnification (right column) the attached macrophages are visible (black arrows). Scale bars represent 200 µm (left column) and 20 µm (right column).

**Figure 6:**
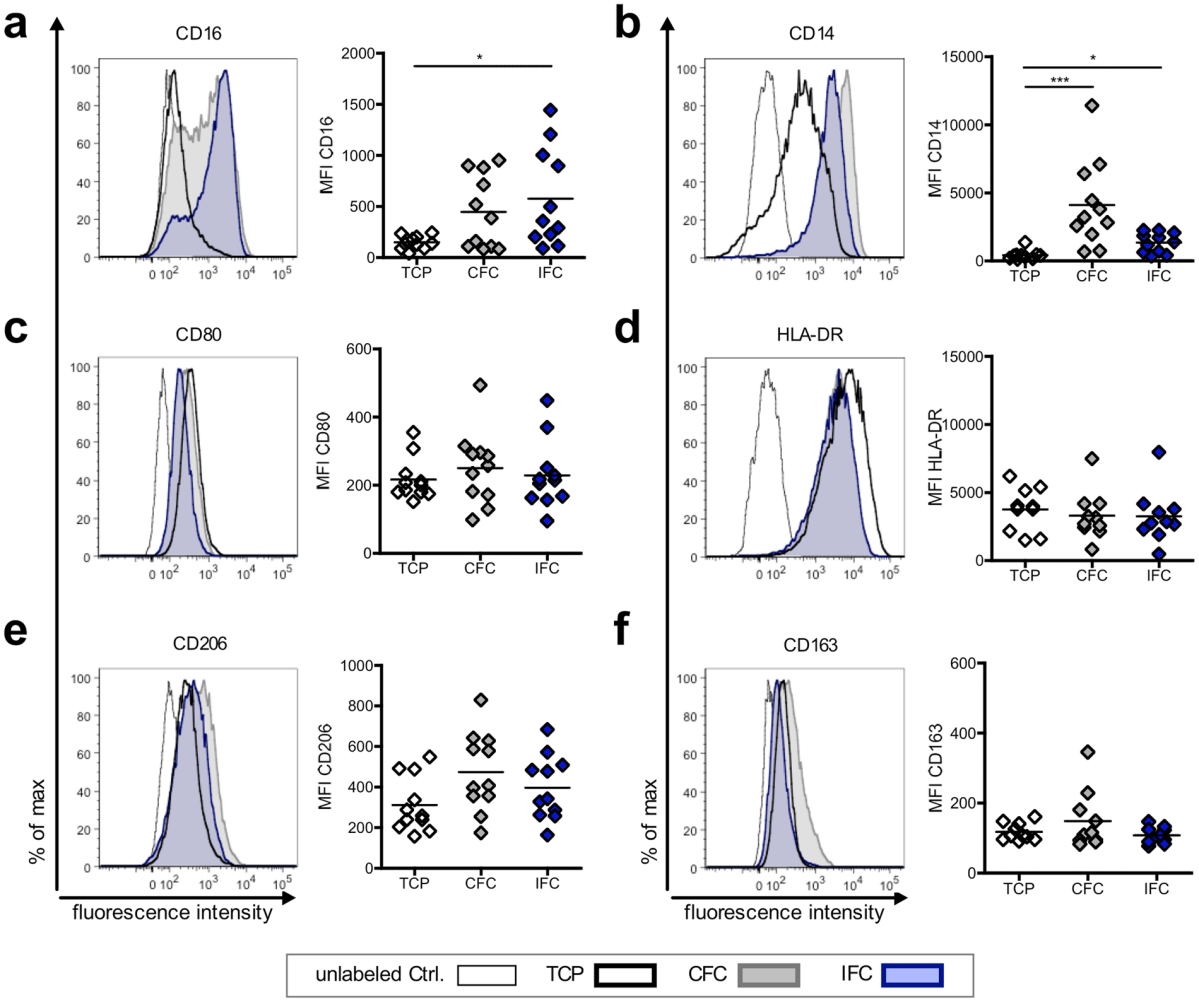
Both types of cryopreserved tissue upregulate common surface molecules on macrophages, but do not influence their polarization state. Macrophages were cultured on the intima surface of CFC or IFC treated aortic tissue. Cells cultured on tissue culture plastic (TCP) served as control. After 2 days, macrophages were harvested, stained with fluorochrome-labeled human specific antibodies and analyzed by flow cytometry. Representative histograms and quantitative analyses of the mean fluorescence intensity (MFI) are shown for the surface markers CD16 (**a**) and CD14 (**b**), the M1 polarization marker CD80 (**c**) and HLA-DR (**d**) and the M2 polarization markers CD206 (**e**) and CD163 (**f**). The means of the data are shown (n = 11) and analyzed with one-way ANOVA (Kruskal-Wallis test) *p < 0.05, ***p < 0.01.

Implanted tissue can affect activation and polarization of resident macrophages that can then drastically influence the immune response. Therefore, we also studied the macrophage responses to CFC and IFC aortic tissue in a newly developed macrophage-tissue assay (Fig. 5). Since macrophages are adherent cells and their features and behavior can be modulated by surface topography, we cultured the macrophages directly on the surface of the aortic tissue. As macrophages originate from monocytes circulating in the blood, we chose the luminal side of the aorta (intima) as the surface for macrophage adherence. Macrophages were differentiated *in vitro* from human blood-derived monocytes by adding M-CSF for 7 days, and then cultivated for 2 days on CFC or IFC human aortic tissue (Fig. 5a). The morphology of the macrophages on the tissue, and the tissue surface itself was examined by scanning electron microscopy (SEM). SEM pictures revealed that macrophages attach to CFC and IFC aortic tissue with similar numbers and morphology (Fig. 5b). Thus, the cryopreservation protocol does not influence the adherence and appearance of macrophages attached to the aortic tissue. However, it is impossible to identify the polarization status of macrophages solely by their morphology, either on the tissue culture plastic or on the tissue itself. Macrophages were harvested after cultivation and their activation and polarization status was determined by flow cytometry. To first exclude potential endotoxin contamination of the human aortic tissue which would influence the macrophage polarization, we tested CFC and IFC tissue samples randomly for pyrogens (method described in Supplementary information). Neither the LAL test, nor the monocyte activation test showed evidence of endotoxin contamination (data not shown). In our previously established *in vitro* macrophage polarization assay, we confirmed the upregulation of the co-stimulatory molecule CD80 and the major histocompatibility complex (MHC) class II molecule human leukocyte antigen (HLA)-DR as clear M1-markers, when macrophages were polarized with IFN-γ and LPS (Supplementary Fig. S5a). A slight upregulation of the mannose receptor CD206 and the scavenger receptor CD163 was observed when macrophages were polarized with IL-4 or IL-10 to M2a or M2c phenotypes, respectively. Consequently, in the macrophage-tissue assay, macrophages were harvested and stained for M1 and M2 polarization markers and other common macrophage surface markers (Fig. 6). A defined gating strategy was used to define single viable cells before the intensity of surface molecule expression was measured (Supplementary Fig. S5b). Interestingly, macrophages cultured on the intimal surface of IFC tissue showed a prominent upregulation of the Fc-gamma receptor CD16, a molecule involved in phagocytic processes, compared to control macrophage cultures on tissue culture plastic (TCP) (Fig. 6a). The common macrophage marker CD14 (LPS receptor) was upregulated on cells cultured on either tissue compared to TCP, whereby macrophages on CFC tissue expressed the highest levels (Fig. 6b). Expression of the M1 polarization markers CD80 and HLA-DR was not changed by cultivation on the tissue itself or by the cryopreservation method applied to the tissue (Fig. 6c,d). A tendency towards increased expression of the M2 polarization markers CD206 and CD163 was observed for cells cultured on CFC tissue, however changes in the mean fluorescence intensity (MFI) were not significant (Fig. 6e,f).

## Discussion

Human cardiovascular grafts need to be processed and stored for clinical use to ensure constant availability and to enhance safety by allowing for quality testing before use. So far, conventional frozen cryopreservation (CFC) is the chosen method for allografts. However, ice-crystal formation destroys or alters the extracellular matrix (ECM)^44^. It was recently shown that the newly developed ice-free cryopreservation (IFC) method prevents undesirable matrix alterations and attenuates immunogenicity^33,43^.

In the present study, we compared the impact of the gold standard method CFC and the alternative IFC method on the biological characteristics of cardiovascular matrices with a specific focus on their immunological compatibility. Our results show that IFC of human aortic tissue clearly modulates human immune responses. We also show strong evidence as to which tissue characteristics and mechanisms are responsible for the observed enhanced tissue immune compatibility.

We clearly demonstrate that the application of IFC for human aortic tissue modifies general tissue characteristics. The aortic tissue still contains cells after both preservation methods, as verified by the presence of nuclei in histological sections as well as an endothelial layer on the intimal side of the tissue. However, the metabolism after IFC was extremely low compared to the CFC tissue from the same tissue donor (see Fig. 1). Similar results were obtained previously for xenogeneic arteries^45^. We hypothesize that the one step process results in water rushing out of the cells in response to the high concentration of cryoprotectants in the VS83 solution. If enough water is removed, full rehydration is impossible for the cells. Therefore, the metabolism of IFC tissue is downregulated and compared to CFC tissue, the levels of apoptosis or necrosis are negligible. Consequently, IFC tissue would act similarly to decellularized tissue. However, preserving the tissue using the VS83 solution is much easier and more effective than complex and extensive decellularization and washing steps. So far, decellularization has been proposed as a preservation method to overcome the problem of immune responses to donor tissues. A wide range of decellularization protocols for cardiovascular tissue exist^46–49^, but the clinical application of decellularized heart valve allografts has led to unsatisfactory outcomes^16,50–52^. Most likely, this is because decellularization destroys or alters the ECM^21^, which would not be the case for the IFC method since it is known that typical matrix structures are preserved^32^.

Another tissue treatment described as “devitalization” by Muratov *et al*. demonstrated similar effects as the IFC method regarding enhanced biocompatibility compared to the conventional cryopreserved tissue^53^. *In vivo* studies of implanted “devitalized” tissues revealed no immune cell infiltration in the tissue after 4 months. Similar findings were observed when IFC heart valves were implanted in a sheep model^31^. IFC tissue was acellular after 7 months of implantation, while CFC tissue showed evidence of fresh cellular T cell infiltration of the acellular matrix. However, in contrast to the IFC method, no deeper investigations regarding the ECM structure have been carried out after the “devitalization” step.

In agreement with the finding that IFC treatment leads to a downregulation of the tissue metabolism, we found that the levels of cytokines and chemokines released were drastically reduced (see Fig. 2). Since there was no increase in the cytokine amounts released from IFC tissue cultures over time, we hypothesize that in IFC tissue, cytokines are no longer produced by cells, but rather any cytokines stored in the ECM are released. In contrast, CFC tissue, which still contains metabolically active cells, secretes especially pro-inflammatory cytokines and chemokines like IL-6 continuously. The reduced cytokine release after IFC treatment would therefore support the *in vivo* performance by reducing the risk of calcification. Calcification is known to be triggered by pro-inflammatory cytokines, especially IL-6, delivered by metabolically active cells of the matrix^54,55^. Surprisingly, we found that although the CFC tissue releases higher total amounts of the cytokine TGF-β, IFC tissue releases more of the active form of TGF-β, though the level is relatively low. TGF-β is synthesized as a pro-peptide bound to the latent TGF-binding protein and targeted to the ECM. TGF-β can be released from this complex by various mechanisms such as conformational changes, binding to integrins, proteolytic cleavage by metalloproteinases, pH changes or oxidative processes^56,57^. Therefore, we speculated that components of the VS83 solution could cause the activation of the TGF-β stored in the ECM. Whereas the 10% DMS0 used in the CFC method did not activate TGF-β, the components of the IFC solution (25% formamide and 36% DMSO) led to higher levels of the active cytokine (see Supplementary Fig. S1). The presence of active TGF-β might influence the interaction with immune cells in different ways. Acting alone, TGF-β is able to skew the differentiation of T cells towards a regulatory cell type (Treg). However, in combination with IL-6, TGF-β drives T cell differentiation towards a T cell subset associated with chronic inflammation, known as Th17 cells^58^. This might be the case for CFC tissue. We hypothesize that the absence of IL-6 combined with low levels of active TGF-β, as occurs after IFC, triggers the generation of Tregs. We also observed low released levels of IL-10 from IFC and CFC tissue that might additionally help to support the development of Tregs. However, we could not verify an enhanced generation of Tregs using an *in vitro* PBMC culture supplemented with conditioned medium from IFC tissue compared to CFC tissue (data not shown). The reduced level of the cytokines released by the IFC tissue clearly dampens the attraction and migration of immune cells, as shown here for monocytes and T cells, compared to a strong attraction towards CFC tissue (see Fig. 3). The reduced release of IL-6 by IFC tissue might lead to enhanced apoptosis of T cells, because of the generation of fewer anti-apoptotic molecules which depend on the cytokine IL-6^59,60^.

Interestingly, we found that the complex cytokine “cocktail”, which is released by the CFC tissue was the strongest trigger for the migration of immune cells, exceeding levels using IL-6 or MCP-1 alone as an attractant (see Supplementary Fig. S3). Importantly, only factors released by the CFC matrix were able to serve as the second co-stimulatory signal during an αCD3 triggered T cell response and amplify the proliferation of both major T cell subsets (see Fig. 4). Besides the role of cytokines in this process, it can be assumed that different types of danger associated molecules (DAMPs) may be involved. For example, DNA fragments, heat shock proteins, and necrotic cell debris among others can enhance immune cell activation via Toll like receptors (TLRs)^36^. TLRs are expressed on antigen presenting cells, but also directly on T cells, and their activation results in modulated immune responses^61,62^. Enhanced release of DAMPs from CFC tissue can be expected owing to the ice-crystal formation process and the higher degree of necrotic cells. In contrast, IFC tissue seems to be unable to deliver additional danger signals since the matrix structure is preserved and the cells appear to be inactive within the matrix. Both the lower level of attraction and migration of immune cells toward the IFC tissue, and the inhibition of T cell proliferation seen *in vitro*, might explain the lower number of infiltrated T cells in allogeneic IFC heart valves after *in vivo* implantation in a sheep model^31^.

In addition, we studied the influence of both cryopreserved matrices on macrophage activation and polarization. It is well known that macrophage activation status plays a crucial role in the outcome of implanted tissues^39,63–66^. Since not only soluble factors influence macrophage activation, but also the surface characteristics of a material, we developed an *in vitro* assay, where macrophages are cultured directly on the surface of the CFC or IFC aortic tissue. In general, the macrophages were viable under these culture conditions and had a similar appearance when cultured on either cryopreserved tissue (see Fig. 5), in contrast to the low viability and unfavorable morphological changes observed when GA fixed tissue was tested as a control (data not shown). This information corroborates with published results that GA fixation of tissue generates dead and structurally deteriorated tissue^6,14,67^ and has a negative impact on the long-term function of cardiovascular grafts^6^. Importantly, we found in our macrophage-tissue assay that neither the IFC nor the CFC-treatment was able to trigger the generation of pro-inflammatory M1-type macrophages (see Fig. 6), as a strong upregulation of the hallmarks of this polarization type, the markers CD80 and HLA-DR, did not occur. Instead, there was a trend towards an increase in the M2-type marker CD206 in co-cultures with either matrix type, which would imply that macrophages would become more of an anti-inflammatory type^39,42^. Macrophages with these characteristics could promote tissue remodeling, remove debris and would lead to a cell free matrix, as seen in an earlier *in vivo* study^31^. The enhanced active TGF-β release detected for IFC matrices might also affect the macrophage function since M2c polarization was linked to higher values of active TGF-β^68,69^. While little is known about the functional consequences of altered CD14 (LPS receptor) and CD16 (Fc gamma receptor) expression on macrophages, we found a rather high expression level of CD16 and a low expression of CD14 by macrophages cultured on IFC tissue compared to CFC tissue. The implications of these alterations require further investigation.

**Figure 7:**
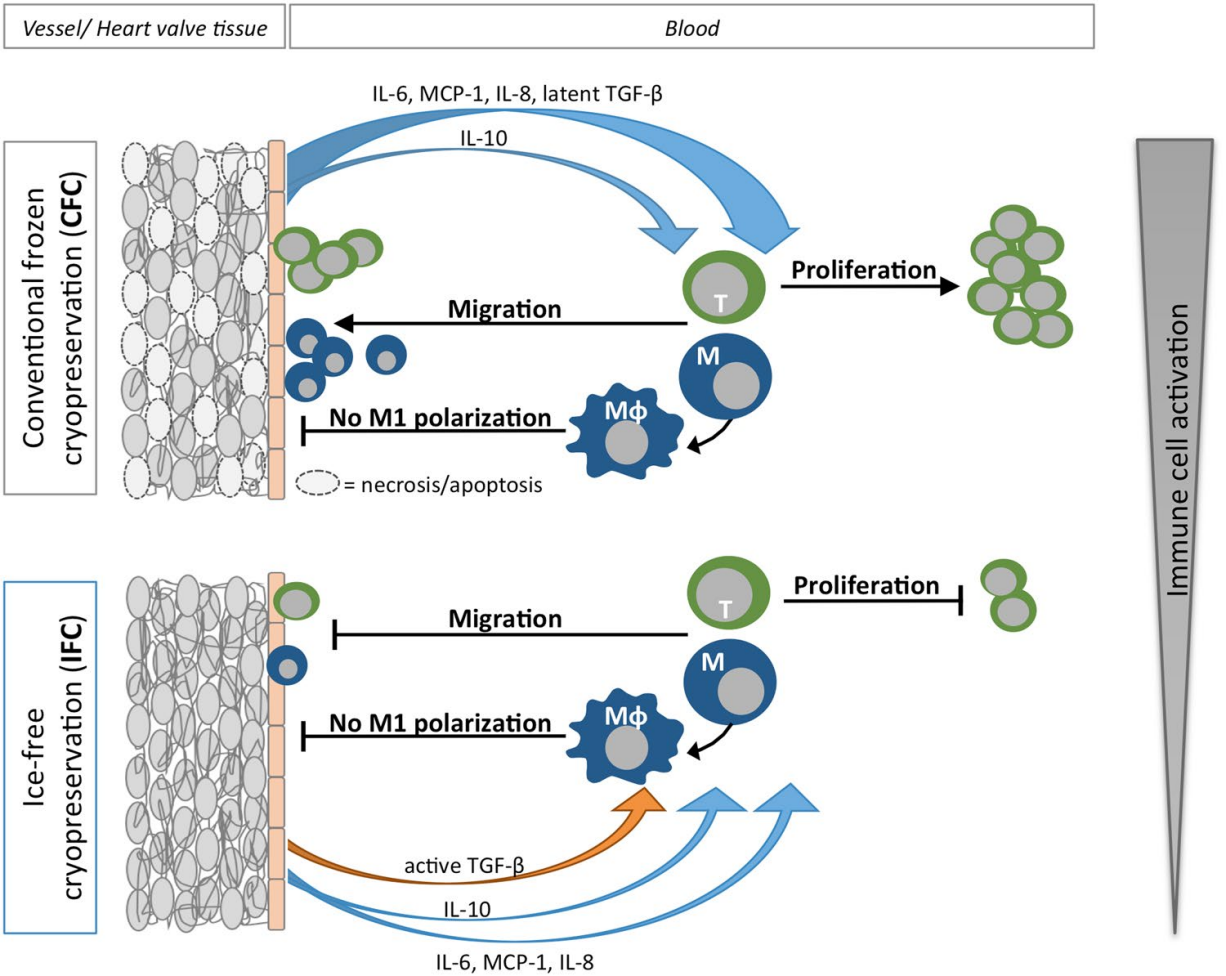
Hypothesized mechanism indicating how the cryopreservation methods affect the immune compatibility of human cardiovascular matrices. Potential effects of CFC and IFC treatments on the human immune response to cardiovascular tissue (vessel or heart valves) are illustrated. After CFC, some tissue cells are apoptotic or necrotic, but the tissue still releases high amounts of cytokines such as IL-6, MCP-1, IL-8, latent TGF-β, and smaller amounts of IL-10. These cytokines lead to a strong activation of blood immune cells. Monocytes and T cells are attracted to the tissue graft, where they can infiltrate or receive signals to proliferate. In contrast, after IFC treatment, tissue cells do not undergo apoptosis or necrosis, but have a diminished metabolic activity. The cytokines IL-6, MCP-1, IL-8, IL-10, and TGF-β are released only in small amounts and TGF-β is secreted in its biologically active form. The decreased levels of cytokine release and the presence of active TGF-β lead to an attenuated activation of immune cells. Particularly, migration and infiltration of T cells and monocytes is reduced and T cell proliferation is blocked, which results in a diminished human immune response to IFC tissue compared to CFC.

In summary, IFC is an easy and cost-effective method, which already showed excellent results in preserving ECM and attenuating inflammation in previous *in vivo* studies. The present study provides more insight into the mechanisms involved in the human immune response towards IFC cardiovascular matrices as illustrated in Fig. 7. Since IFC led to reduced immunogenicity of the tissue compared to CFC, it is a promising new possibility for clinical preservation and storage of cardiovascular allografts.

## Conclusion

We showed that IFC reduces tissue metabolism and the level of apoptosis/necrosis of the remaining cells, as well as decreases the amount of pro-inflammatory cytokines and chemokines released. Because of these modified tissue characteristics, the innate and adaptive immune responses are dampened, leading to lower immunogenicity of the cardiovascular transplants after IFC treatment.

## Materials and Methods

More details about the materials and methods section are attached as Supplementary information in the online version of this paper.

### Tissue preparation, cryopreservation and rewarming

Human aortic tissue was obtained from patients undergoing replacement of the ascending aorta at Deutsches Herzzentrum Berlin (Germany). The collection of tissue during the surgical procedure was approved by the ethics committee of Charité - Universitätsmedizin Berlin (EA4/028/12) and all patients gave written informed consent. Aortic tissue was washed, treated with antibiotics and frozen as described elsewhere^33^. The volumes of the original protocol were modified, since for all experiments 8 mm tissue punches were made using a biopsy punch (pfm medical, Köln, Germany). Briefly, tissue punches were frozen according to conventional frozen cryopreservation (CFC) protocol with CFC-medium (pyruvate-free Dulbecco’s Modified Eagle Medium with glutamine, 4.5 g/L glucose, 3.7 g/L NaHCO3 (DMEM; Biochrom, Berlin, Germany) containing 10% human albumin (200 g/L infusion solution; Baxter, Unterschleiβheim, Germany) and 10% dimethyl sulfoxide (DMSO; Sigma-Aldrich, St. Louis, MO, USA) for 1 h on ice or according to ice-free cryopreservation (IFC) protocol with a cryoprotectant solution designated VS83 (Euro-Collins solution containing 4.65 mol/L formamide, 4.65 mol/L DMSO, and 3.31 mol/L 1,2 propanediol (all Sigma-Aldrich)) for 1 h at room temperature (RT). The detailed protocol can be found in the Supplementary information. All experiments were performed in accordance with relevant guidelines and regulations.

### Immunohistology for endothelial cell detection

CFC or IFC treated human aortic tissue was embedded in tissue freezing medium (Leica, Nussloch, Germany). Six µm cryosections were stained for CD31. Briefly, after blocking with PBS containing 1% bovine serum albumin and 5% donkey serum (both Sigma-Aldrich), and washing with PBS (Biochrome), the mouse-anti-human CD31 antibody (1:50; eBioscience/Thermo Fisher Scientific, Waltham, MA, USA) incubated overnight at 4 °C and was detected bya donkey-anti-mouse IgG Alexa Fluor 555 antibody (1:50; Thermo Fisher Scientific). Sections were counterstained with 4′,6-diamidino-2-phenylindole (DAPI) mounting medium (Dianova, Hamburg, Germany). Pictures were taken with the Operetta High-Content Imaging System (Perkin-Elmer, Waltham, MA, USA) at 20x magnification. Image analysis was performed with the Columbus™ Image Data Storage and Analysis System (Perkin Elmer).

### TdT-mediated dUTP nick-end labeling (TUNEL) tissue staining

A modified colorimetric TUNEL assay (Promega, Mannheim, Germany) was used to detect apoptotic cells. Tissue cryosections were stained according to the manufacturer’s instructions. As a positive control, tissue slides were treated with DNAse I (Macherey-Nagel, Düren, Germany). Pictures were taken using a DMi8 microscope (Leica, Nussloch, Germany) with 10x magnification.

### Cell viability assays

Metabolic activity of aortic tissue was assessed using an assay based on the reduction of 3-(4,5-dimethylthiazol-2-yl)-5-(3-carboxymethoxyphenyl)-2-(4-sulfophenyl)-2H-tetrazolium (MTS) by viable cells with CellTiter 96^®^ AQ_ueous_ One Solution Assay (Promega). Tissue punches were incubated in DMEM overnight at 37 °C. Medium was exchanged before MTS assay was performed according to the manufacturer’s protocol. Absorbance at the 490 nm wavelength was measured (SpectraMax 340PC; Molecular Devices, Sunnyvale, CA, USA).

Necrotic cells or cells with membrane damage in aortic tissue were determined by a lactate dehydrogenase (LDH) release assay (CytoTox-ONE™ Homogeneous Membrane Integrity Assay; Promega). Tissue punches were incubated in DMEM for 1 h at 37 °C before assay was performed according to the manufacturer’s protocol. Fluorescence was read with excitation at 560 nm and emission at 590 nm (Infinite 200 PRO, Tecan, Männedorf, Switzerland).

Caspase 3/7 activity was determined using Caspase-Glo^®^ 3/7 assay (Promega). Tissue punches were incubated in DMEM for 1 h at 37 °C before assay was performed according to the manufacturer’s protocol. After 1 h incubation, luminescence was measured (Mithras LB 940, Berthold Technologies, Bad Wildbad, Germany).

### Cytokine detection

CFC and IFC human aortic tissue punches were incubated in DMEM for 1–6 days at 37 °C. Supernatants were taken and analyzed for human interleukin (IL)-10, monocyte chemoattractant protein (MCP)-1, IL-6, and transforming growth factor (TGF)-β1 using an enzyme-linked immunosorbent assay (ELISA; ELISA MAX™ Deluxe; BioLegend, San Diego, CA, USA) according to the manufacturer’s protocol. IL-8, tumor necrosis factor (TNF)-α, interferon (IFN)-γ, IL-1β and IL-12p70 were evaluated using a multiplex bead-based assay (LEGENDplex™, BioLegend) according to the manufacturer’s protocol. For the kinetic analysis (day 1–6), supernatant was taken every day from the same culture well and the cytokine concentration was back calculated to the remaining volume of the culture medium to obtain the absolute cytokine amount (in pg) produced per tissue punch. Absorbance of ELISA samples was measured at 450 nm on a plate reader (SpectraMax). Multiplex samples were measured by flow cytometry using the BD FACS-Canto II (BD Biosciences, San Jose, CA) and analyzed with LEGENDplex™ version 7.1 (VigeneTech Inc, Carlisle, MA, USA).

### Immune cell isolation and purification

Peripheral blood mononuclear cells (PBMC) were isolated from buffy coats (German Red Cross, Berlin, Germany) or from healthy volunteers with written informed consent using protocols approved by the ethics committee of the Charité - Universitätsmedizin Berlin (EA1/226/14; EA2/139/10) using a Biocoll gradient as described previously^70^. All experiments were performed in accordance with relevant guidelines and regulations.

Monocytes (CD14+ cells) were enriched from the PBMC fraction using magnetic activated cell sorting (MACS). PBMC were incubated with CD14 MicroBeads and isolated according to the manufacturer’s protocol with LS Columns (all MACS material from Miltenyi Biotec, Bergisch Gladbach, Germany).

T cells (CD3+ cells) were separated from the CD14-negative MACS fraction using CD3 MicroBeads (Miltenyi Biotec). The purity of separated cells was confirmed by staining with the human specific antibodies CD14-APCCy7 or CD3-PerCPCy5.5 (BioLegend). FACS staining and measurement at FACS Canto II was performed as described elsewhere^32^. Purity ranged between 95–99%.

### Immune cell migration assay

Conditioned medium (CM) from CFC or IFC tissues or control medium, was placed in the lower well of a 3 µm pore size chemotaxis system (ChemoTx^®^ Disposable Chemotaxis System; Neuro Probe, Gaithersburg, MD, USA). Diet-medium (very low endotoxin Roswell Park Memorial Institute 1640 culture medium (RPMI; Biochrom) with 1% human serum from male AB plasma (AB-serum; Sigma-Aldrich), 1% penicillin/streptomycin (P/S) and 1% glutamine (both from Life Technologies, Carlsbad, CA, USA) alone served as a negative control to define random cell migration without a chemotactic gradient. CFC and IFC tissue CM was generated by incubating a tissue punch for 6 days in diet-medium. Positive control settings are described in the Supplementary information. Freshly purified monocytes (CD14+) and T cells (CD3+) were resuspended in diet-medium. Thirty thousand cells were seeded on the upper side of the membrane. After 3 h at 37 °C, cells that migrated to the lower well were counted with a Fuchs-Rosenthal counting chamber. The membrane was fixed and stained with the Hemacolor^®^ Rapid staining kit (Merck) after removing non-migrated cells from the upper side of the membrane with a cotton bud. Pictures of the bottom side of the membrane were taken with an Axio Scope microscope (Zeiss, Jena, Germany) and the images were processed using Fiji-ImageJ software^71^ to count additional cells attached to the bottom side of the membrane and obtain the total number of migrated cells.

### Immune cell proliferation assay

PBMC were labeled with carboxyfluorescein succinimidyl ester (CFSE; Molecular Probes/ Thermo Fisher Scientific) as described elsewhere^33^. 3 × 10^5^ labeled PBMC were seeded in 400 μL complete RPMI (RPMI with 10% human AB-serum, 1% P/S and 1% glutamine) in 48-well plates. To induce a basal level of activation, PBMC were stimulated with a low dose (12.5 ng/mL) of αCD3 antibody (OKT3; Janssen-Cilag, Neuss, Germany). CFC and IFC tissue CM was made by incubating 8 mm tissue punches for 6 days in complete RPMI. Four hundred µL of CFC- or IFC-CM was added to the αCD3 stimulated PBMC culture. PBMC cultures without αCD3 stimulation served as control. After 4 days culture, PBMC were harvested with trypsin (Gibco/Thermo Fisher Scientific) and stained with human specific antibodies: CD8-PE (1:100) (Miltenyi Biotec), CD4-APC (1:100), CD14-V450 (1:1000) (BD Biosciences) and a viability marker in the V510 channel (1:400) (LIVE/DEAD® Fixable Aqua Dead Cell Stain Kit; Invitrogen/Thermo Fisher Scientific). FACS staining and measurement was performed as described above.

### Macrophage-tissue assay

Purified human monocytes were thawed and cultured in complete RPMI at a density of 2 × 10^6^ cells per well of a 6-well plate containing 50 ng/mL macrophage colony-stimulating factor (M-CSF; Miltenyi Biotec) for 7 days. Differentiated macrophages (M0) were harvested with a cell scraper and washed with PBS. In 200 µL complete RPMI, 1 × 10^5^ cells were seeded directly onto the intimal surface of the CFC or IFC human aortic tissue punch which had been frozen and rewarmed as described above. To ensure direct contact of the macrophages with the tissue the punch was held to the bottom of the 48-well plate with a small ring cut from a silicone tube (Ismatec/Cole-Parmer, Wertheim, Germany). After 2-day co-cultures at 37 °C, macrophages were harvested from the tissue surface with Accutase (Gibco/Thermo Fisher Scientific) and stained with the human specific antibodies CD163-FITC (1:20), CD80-PE (1:20), CD16-PerCPCy5.5 (1:200), CD206-APC (1:100), HLA-DR-PECy7 (1:400) (all BioLegend), CD14-APCCy7 (1:100) (BD Biosciences) and a viability marker in the V450 channel (1:1000) (LIVE/DEAD® Fixable Violet Dead Cell Stain Kit; Invitrogen/Thermo Fisher Scientific). FACS staining and measurement was performed as described above.

### Scanning electron microscopy (SEM)

Macrophages were cultured for 2 days on aortic tissue. Without harvesting the macrophages, tissue punches were washed with PBS and fixed with 2.5% grade I glutaraldehyde (Sigma-Aldrich) for 10 min. A series of ethanol (Carl Roth, Karlsruhe, Germany) concentrations in distilled water were applied to dehydrate the tissue. Accordingly, tissue punches were successively incubated for 5 min each in 30%, 50%, 70%, 80%, 90%, 95% ethanol, and twice in 100% ethanol. Finally, drying of the sample was performed using 3 incubations for 10 min with hexamethyldisilazane (HMDS; Sigma-Aldrich). Tissue samples were sputter coated with gold for 30 s (JFC-1200 Fine Coater; JOEL, Freising, Germany). Pictures were taken with the JCM 6000 benchtop SEM (JOEL) in high vacuum mode at 10 kV.

### Statistical methods

Results are depicted as the mean + standard error of the mean (SEM). Statistical differences between two groups were analyzed using the non-parametric Mann-Whitney test. For more than two groups, the Kruskal-Wallis non-parametric one-way analysis of variance (ANOVA) was used with Dunn’s post test. Differences between groups were considered significant with *p < 0.05, **p < 0.01, and ***p < 0.001. Statistical analysis was performed using GraphPad Prism version 6.0 software (GraphPad Software, San Diego, CA, USA).

### Data Availability

The datasets generated and/or analyzed during the current study are available from the corresponding author on reasonable request. MSch and MS have access to all the data.

## Electronic supplementary material

Supplementary information
