# Supplementary material for: The choice of cryopreservation method affects immune compatibility of human cardiovascular matrices
Source: Sci Rep. 2017 Dec 5;7:17027. doi: 10.1038/s41598-017-17288-z (PMC5717054; doi:10.1038/s41598-017-17288-z)
Supplement: Supplementary file 1 — Supplementary information [file 41598_2017_17288_MOESM1_ESM.pdf]

Supplementary information

The choice of cryopreservation method affects immune compatibility of human cardiovascular matrices

Short title: Cryopreservation strategy affects immune compatibility

Maria Schneider[1,2], Christof Stamm[2,3], Kelvin G.M. Brockbank[4,5], Ulrich A. Stock[6], Martina Seifert[1,2]

1. Study design – underlying concept of the two preservation methods

We compared two cryopreservation methods, the CFC method that promotes cell viability versus the IFC method that promotes ECM retention and loss of cell viability. The CFC heart valve method was selected because it is the most commonly used method of human heart valve cryopreservation in clinical use. Development of ice-free cryopreservation by vitrification methods for heart valves was initially stimulated by demonstration of large ice crystal domains within CFC heart valve tissues employing a method called cryosubstitution [25].The first heart valve vitrification studies utilized a vitrification solution (VS) called VS55 to reflect its 55% (w/v) of cryoprotectant solutes. VS55 consists of an 8.4 M mixture of 1,2- propanediol, formamide, and dimethyl sulfoxide in Euro-Collins (EC) solution. However, upon scale up from small parts of heart valves, 1-3 mL volume, to intact full-sized heart valves, 80–100 mL volume, two problems were encountered: (1) cracking at vapor-phase nitrogen temperatures, particularly in dry nitrogen shippers, and (2) the VS55 solution demonstrated ice formation during rewarming [27]. The solution to both problems was to increase the concentrations of the three cryoprotectants in VS55 from 55 to 83 % to make VS83. Furthermore, modulated differential scanning calorimetry studies indicated that this new formulation was free from ice and potentially stable above the glass transition temperature of VS83 at −80°C [27], which would make it easier and cheaper to store and ship the tissue samples. Storage at −80°C was subsequently incorporated into the IFC method.

Other differences between the cryopreservation protocols include the cryoprotectant addition and removal strategies. The CFC method and the original IFC VS55 method

emphasized preservation of cell viability so cryoprotectants were added on ice and stepwise methods used to minimize the risks of osmotic shock. Mannitol was also used in the washout solutions to minimize osmotic shock to the cells. In the IFC method evolution from VS55 to VS83 cryoprotectant solutions efforts to retain cell viability were given up [27]. Furthermore, the IFC protocol was subsequently modified to promote loss of cell viability by using a single step room temperature cryoprotectant loading strategy and washout protocol without any attempts at osmotic buffering to retain cell viability [43]. Cooling is probably not necessary for the VS83 effects observed here because post-thaw-treatment of CFC human heart valves with VS83 demonstrated improved immune compatibility [33]. Further study of fresh heart valve tissue without cooling is needed. The evolution of the IFC process employed here has been reviewed in depth [71].

2. Methods in detail

Tissue preparation, cryopreservation and rewarming

After explantation, the aortic tissue was washed twice in cold Ca^{2+} and Mg^{2+}-free phosphate buffered saline (PBS; Biochrom, Berlin, Germany) for 5 min and then treated with an antibiotic cocktail containing 15 mg/L Amikacin, 37.5 mg/L Flucytosine, 15 mg/L Vancomycin, 3.75 mg/L Ciprofloxacin, and 15 mg/L Metronidazole in pyruvate-free Dulbecco's Modified Eagle Medium with glutamine, 4.5 g/L glucose, 3.7 g/L NaHCO3 (DMEM; Biochrom, Berlin, Germany) with 10% human serum from male AB plasma (AB-serum; Sigma-Aldrich, St. Louis, MO, USA) for 24 h at 4°C. After antibiotic treatment, 8 mm tissue punches were made using a biopsy punch (pfm medical, Köln, Germany). Tissue punches were frozen according to either the conventional or the ice-free cryopreservation protocol with random allocation. All solutions and media were filter sterilized using 0.2 µm filter systems (Merck, Darmstadt, Germany) before application.

Conventional frozen cryopreservation (CFC) of tissue punches was done by incubating each punch in 5 mL CFC-medium (DMEM containing 10% human albumin (200 g/L infusion solution; Baxter, Unterschleiβheim, Germany) and 10% dimethyl sulfoxide (DMSO; Sigma-Aldrich, St. Louis, MO, USA) for 1 h on ice. Thereafter, the punch was transferred into a 1.8 mL cryo tube (Sarstedt, Nümbrecht, Germany) with

CFC-medium and frozen at a controlled rate of -1°C per min to -80°C (Kryo560 controlled rate freezer; Planer PLC, Middlesex, UK). The tissue punches were then stored in the vapor phase above liquid nitrogen (about -160°C) for at least 1 month.

Ice-free cryopreservation (IFC) of tissue punches was achieved by incubating each punch in 5 mL of a 83% cryoprotectant solution designated VS83 (Euro-Collins solution [174.76 g/L dextrose, 10.2 g/L KH2PO4, 36.5 g/L K2HPO4, 5.6 g/L KCL, 4.2 g/L NaHCO3] containing 4.65 mol/L formamide, 4.65 mol/L DMSO, and 3.31 mol/L 1,2 propanediol (all Sigma-Aldrich, St. Louis, MO, USA)) for 1 h at room temperature (RT) as previously described [42], [43]. Afterwards, the punch was transferred into a 1.8 mL cryo tube with VS83 solution and cooled down by placing the tube in a bath of precooled (-135°C) 2-methylbutane (Sigma-Aldrich, St. Louis, MO, USA). Cooled tissue punches were stored in a -80°C freezer for at least 1 month.

For later analysis and immunological tests, tissue was rewarmed by placing the cryo tubes in a 37°C water bath until ice was thawed. CFC tissue punches were washed for 5 min each in 5 mL 4°C cold DMEM with 0.5 mol/L mannitol (Sigma-Aldrich, St. Louis, MO, USA) followed by DMEM with 0.25 mol/L mannitol and then DMEM alone. IFC tissue punches were washed 5 times with Euro-Collins solution at RT for 5 min.

Endotoxin test

The endotoxin load of human aortic tissue was randomly tested using a limulus amoebocyte lysate (LAL) chromogenic endotoxin quantitation test (Pierce; Thermo Fisher Scientific) or by performing a monocyte activation test (MAT). For both tests, CFC or IFC human tissue punches were incubated and shaken in DMEM medium overnight. Afterwards, the LAL test was performed by testing the conditioned medium (CM) according to the manufacturer’s protocol. For the MAT assay, 1×10^6 PBMC were seeded per well of a 24-well plate in RPMI and treated with CFC- or IFC-tissue CM. PBMC stimulated with 100 ng/mL LPS served as a positive control. After 24 h, supernatants were taken and the amount of TNF-α produced by the PBMC was measured by ELISA (BioLegend). Absorbance was measured at 450 nm using a plate reader (SpectraMax). The TNF-α level correlates with endotoxin contamination and samples were considered low in endotoxin when TNF-α was less than the TNF-α level of corresponding 10 ng/mL LPS positive control.

Migration assay positive controls

For migration assay controls (Fig. S3), The positive control medium was generated by 7-day αCD3 (OKT3; Janssen-Cilag, Neuss, Germany) and αCD28 (BioLegend) stimulation of a PBMC culture in complete RPMI (RPMI with 10% human AB-serum, 1% penicillin/streptomycin (P/S) and 1% glutamine (both from Life Technologies, Carlsbad, CA, USA)). Further positive controls included 50 ng/mL MCP-1 or IL-6 (both Miltenyi Biotec) in diet-medium.

Macrophage polarization assay

Human monocytes (CD14+ cells) were thawed and cultured in complete RPMI at $2x10^6$ per well of a 6-well plate containing 50 ng/mL macrophage colony-stimulating factor (M-CSF; Miltenyi Biotec) for 7 days in a humidified atmosphere with 5% CO2 at 37°C. Differentiated macrophages (M0) were harvested with a cell scraper and washed with PBS before being reseeded at $3x10^5$ per well of a 48-well plate in complete RPMI. Macrophages were polarized for 2 days with either 20 ng/mL IFN-γ (Miltenyi Biotec) and 100 ng/mL Lipopolysaccharide (LPS; Escherichia coli O127:B8; Sigma-Aldrich) to obtain an M1 phenotype, or with either 20 ng/mL IL-4 or 20 ng/mL IL-10 (both Miltenyi Biotec) to obtain an M2a or M2c phenotype, respectively. Macrophages were harvested with Accutase, stained with fluorochrome-labeled antibodies and analyzed by flow cytometry (Fig. S5).

3. Figures

Supplementary 1

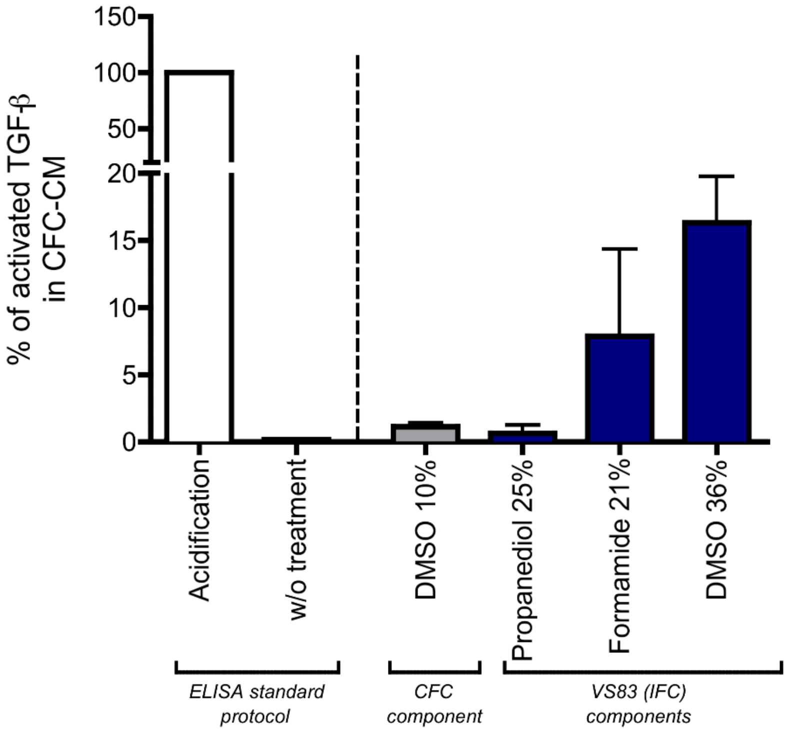

Figure S1: Components of VS83 (IFC) solution partially activate latent TGF-β. CFC tissue conditioned medium (CM) was analyzed using TGF-β ELISA. For activation of latent bound TGF-β, an acidification treatment is necessary. The amount of activated TGF-β with acidification treatment was set as 100%. Without (w/o) any further treatment, 0.2% active TGF-β was detectable. Treatment of the same CFC-CM with 10% DMSO activated 1% TGF-β. Treating CFC-CM with 25% propanediol, 21% formamide or 36% DMSO activated 0.6%, 8% or 16% of the latent bound TGF-β, respectively. Data are shown as the mean + SEM (n=3).

Supplementary 2

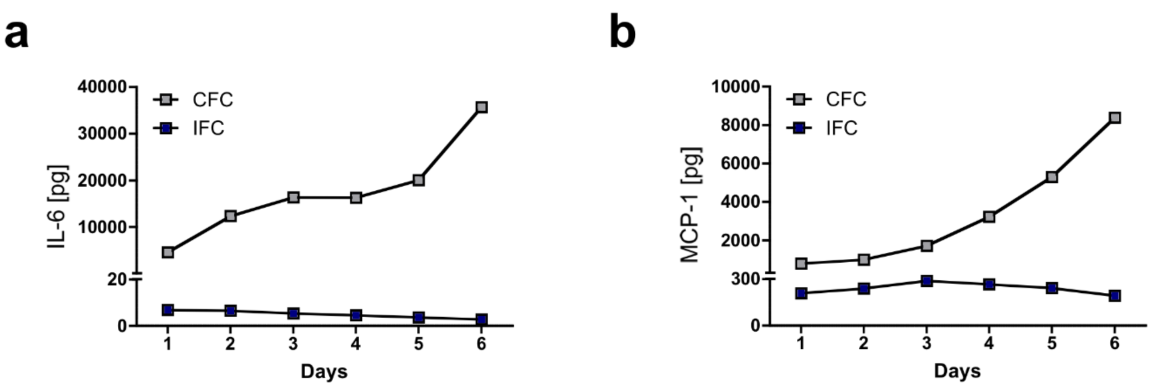

Figure S2: IL-6 and MCP-1 accumulate in CFC tissue culture over time. CFC and IFC aortic tissue punches were incubated in DMEM culture medium for 6 days. The absolute amounts of IL-6 **(a)** and MCP-1 **(b)** were analyzed each day by ELISA, and representative kinetic graphs are shown.

Supplementary 3

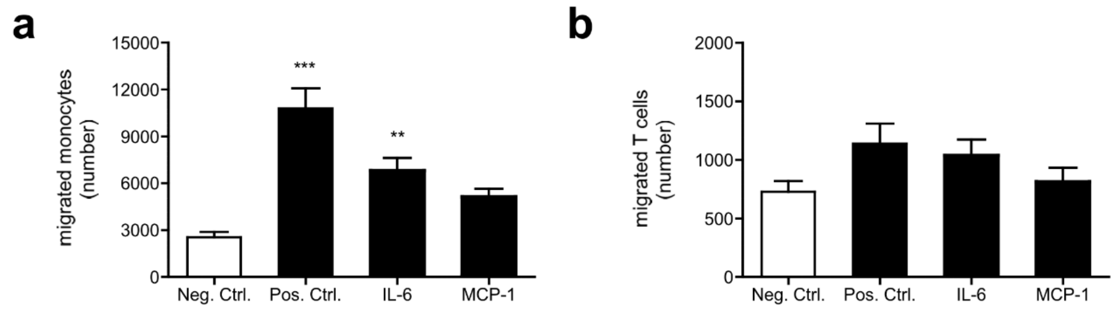

Figure S3: Control settings for immune cell migration assay. Monocytes (CD14+) and T cells (CD3+) were separated from human PBMC. Thirty thousand cells in diet-medium were seeded on the porous membrane of a chemotaxis system. After 3 h, the numbers of migrated monocytes **(a)** and T cells **(b)** were analyzed. Migrated cells were defined as cells in the lower well and attached to the bottom side of the membrane. The supernatant from an αCD3/αCD28 stimulated PBMC culture served as a positive control (Pos. Ctrl.), and 50 ng/mL IL-6 or MCP-1 were further positive controls. Diet-medium alone served as negative control (Neg. Ctrl.), to define the random cell migration without a chemotactic gradient. Data are shown as the mean + SEM (n=5, 3 replicates each) and analyzed with one-way ANOVA (Kruskal-Wallis test) $^{**}p<0.01$, $^{***}p<0.001$ vs. Neg. Ctrl.

Supplementary S4

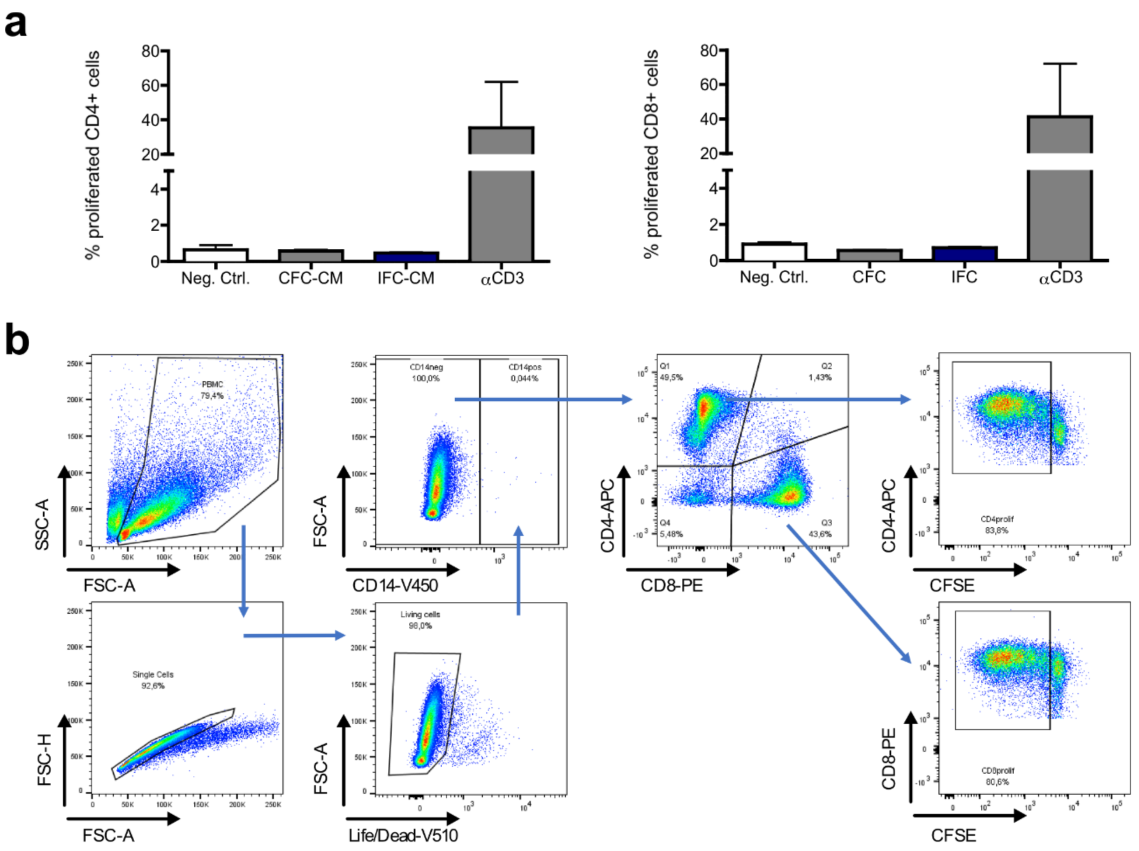

Figure S4: T cell proliferation induction assay. Human CFSE-labeled PBMC were stimulated with CFC or IFC tissue conditioned medium (CM). A low dose of αCD3 antibody served as a positive control and cell culture medium was used as a negative control (Neg. Ctrl.). After 4 days, PBMC were harvested, stained with fluorochrome-labeled human specific antibodies for T cell subset markers and analyzed by flow cytometry. Proliferation rates for CD4+ T cells **(a, left)** and CD8+ T cells **(a, right)** are shown. Data are shown as the mean + SEM (n=3) and analyzed with one-way ANOVA (Kruskal-Wallis test) *$p<0.05$. **b)** FACS gating strategy for T cell proliferation: PBMC were pre-gated via forward scatter area (FSC-A) and sideward scatter area (SSC-A) to exclude cell debris from the analysis. Doublets were excluded by gating on FSC-A vs. forward scatter height (FSC-H). Viable cells were identified (Live/dead vs. FSC-A) and CD14+ cells were gated out (FSC-A vs. CD14). The CD14 negative population (T cells) was further divided into CD4+ vs CD8+ populations. T cell proliferation was measured by CFSE dilution of CD4+ or CD8+ T cells (CFSE vs. CD4 or CFSE vs. CD8), respectively. The gating strategy is shown here for αCD3-stimulated PBMC.

Supplementary 5

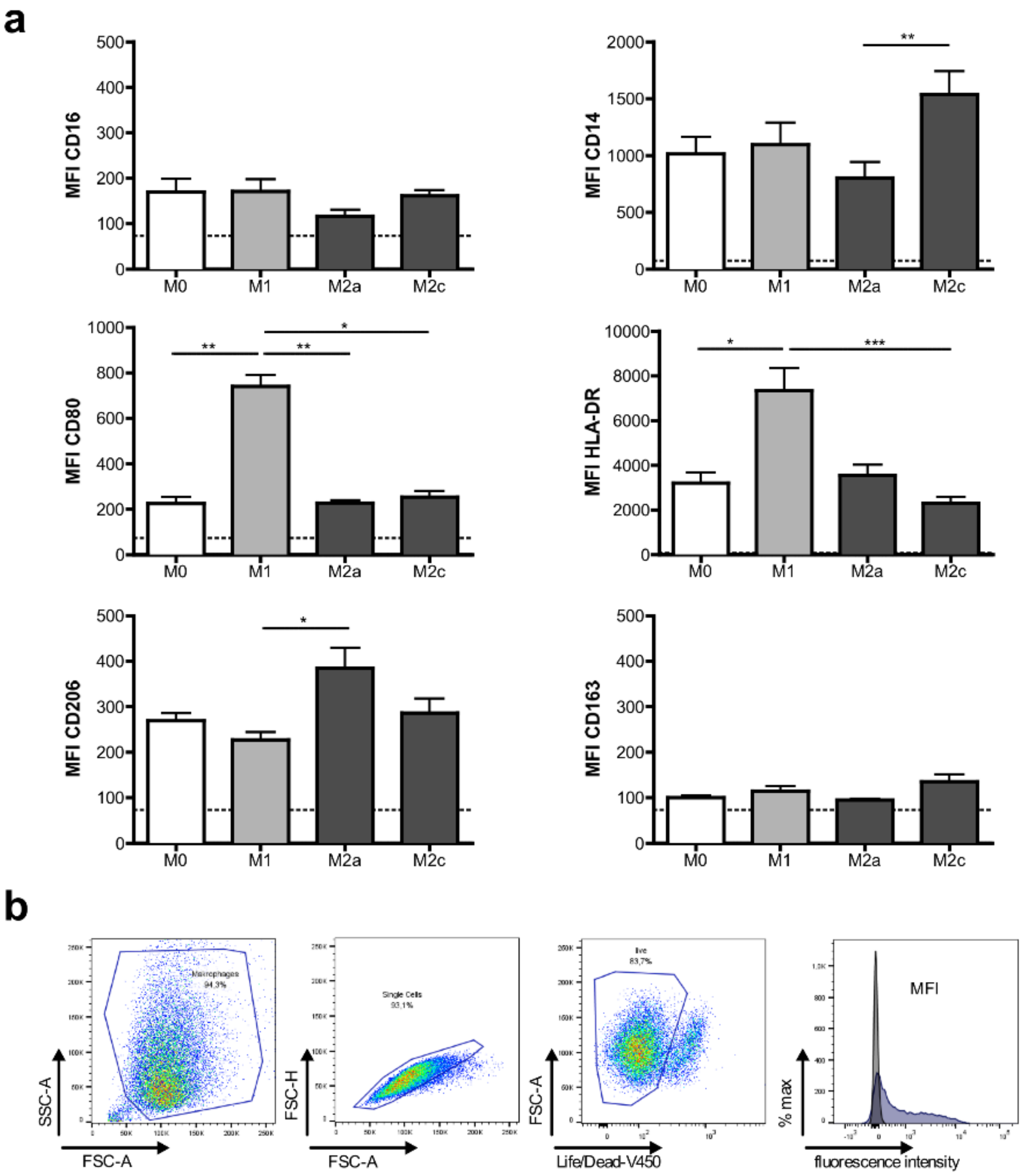

Figure S5: Macrophage polarization assay. Monocyte-derived macrophages were cultured on tissue culture plastic and were stimulated with LPS and IFN-γ to obtain an M1 phenotype, and with IL-4 or IL-10 to obtain M2a or M2c phenotypes, respectively. **a)** Mean fluorescence intensity (MFI) values for the macrophage surface markers CD16, CD14, CD80, HLA-DR**,** CD206 and CD163 are shown for the different polarization phenotypes. Dotted lines in each graph represent the unstained control (background). Mean + SEM of the data is shown (n=7-9) and analyzed with one-way ANOVA (Kruskal-Wallis test) *p<0.05, **p<0.1, ***p<0.01. **b)** FACS gating strategy for surface marker analysis: macrophages were pre-gated via forward scatter area (FSC-A) and sideward scatter area (SSC-A) to exclude cell debris from the analysis. Doublets were excluded by gating on FSC-A vs. forward scatter height (FSC-H). Viable cells were identified (FSC-A vs. Live/Dead) and the MFI of the marker of interest was determined.
